# Anxiety and Interpretation of Ambiguity in Autistic Children, Typical Children and Their Mothers

**DOI:** 10.1007/s10803-018-3781-9

**Published:** 2018-11-08

**Authors:** Louise Neil, Hannah White, Katy Warren, Elizabeth Pellicano

**Affiliations:** 1 Centre for Research in Autism and Education (CRAE), UCL Institute of Education, University College London, London, UK; 2 Present Address: Department of Clinical, Educational, and Health Psychology, University College London, 26 Bedford Way, London, WC1H 0AP UK; 3 Present Address: Department of Educational Studies, Macquarie University, Sydney, Australia

**Keywords:** Autism, Child, Anxiety, Cognition

## Abstract

Anxiety is highly prevalent in autistic children. Yet interpretation biases implicated in anxiety in non-autistic individuals have received little research attention in this group. Twenty-two autistic children and 25 typical children completed an ambiguous scenarios interview and questionnaire-based measures of anxiety. A subsample of mothers completed parent-report and adult relevant versions of the interview and anxiety questionnaires. Autistic children self-reported similar interpretations of ambiguous scenarios, and similar levels of anxiety, to their typical peers. In contrast, mothers of autistic children reported greater levels of anxiety, and more negative interpretations of ambiguous scenarios in both their children and themselves, relative to mothers of typical children. These data highlight the importance of including autistic children’s self-reports when measuring and treating anxiety.

Anxiety, characterized by fearful emotion, worried thoughts and physiological symptoms such as muscle tension, is common in children. Estimates suggest between 6.5 and 10% of children and adolescents are affected by an anxiety disorder (Costello et al. 2003; Polanczyk et al. 2015) and a further 15% to one-third of children are impaired to a mild-to-moderate degree by anxiety symptoms (Angold et al. 1999; Chavira et al. 2004). Autistic children are even more likely to experience anxiety than their non-autistic peers (Merikangas et al. 2010), with as many as 40% (Simonoff et al. 2008; Van Steensel et al. 2011) reaching criteria for a co-occurring anxiety disorder. Autistic children also report more anxiety symptoms in their day-to-day lives than non-autistic children of similar age and ability (Boulter et al. 2014). Research into the beliefs, assumptions and thinking styles which may characterize, generate or sustain anxiety in these children is, however, remarkably limited.

## Anxiety in Non-autistic Children

Anxiety, which can be specific to certain events and situations (e.g., separation anxiety, phobias, social anxiety) or pervasive to several events or activities (in the case of generalized anxiety), is characterized by both hyper-vigilance and avoidance. As well as being distressing in itself it can impact children’s school performance and social functioning (La Greca and Landoll 2011; Wood 2006) run a chronic or recurrent course (Keller et al. 1992; Kessler et al. 2005), and predict the development of mood disorders (Ormel et al. 2015). Fortunately, there is evidence that anxiety disorders in youth can be successfully treated, for example via cognitive behavioural therapy (Cartwright-Hatton et al. 2004), which has been shown to work even in young children (Hirshfeld-Becker et al. 2011). According to cognitive theory, anxiety is characterized by hypervalent negative schemata, which skew interpretations of incoming information towards threat and vulnerability (Beck et al. 1985). Accordingly, individuals with high levels of anxiety and worry show attentional biases towards threat cues in their environment (Hirsch et al. 2011; Macleod et al. 1986; Mogg et al. 1992) and interpretative biases, characterized by perceptions of increased threat (Barrett et al. 1996; Creswell et al. 2005; Muris et al. 2000) and predictions of poor coping (Creswell and O’Connor 2011; Waters et al. 2008). Numerous studies have linked interpretation biases to anxiety in children, most commonly by assessing their responses to hypothetical ambiguous scenarios (Barrett et al. 1996; Creswell et al. 2005, 2011; Lau et al. 2013; Waite et al. 2015). Although there are some studies that have failed to find an association between anxiety and threat interpretation in children (Creswell et al. 2014; Waters 2008), a recent meta-analysis (Stuijfzand et al. 2017) found a medium positive association (*d* = .62) between anxiety and negative interpretations in children and adolescents. Furthermore, a reduction in children’s threat interpretations have been demonstrated following treatment for child anxiety (Creswell et al. 2005).

Environmental factors are likely to play a substantial role in the etiology of anxiety disorders, with heritability estimates between .3 and .67 (Eley and Gregory 2004; Hettema et al. 2001; Spatola et al. 2007). Longitudinal research suggests negative life events (Broeren et al. 2014) and peer victimisation (Siegel et al. 2009) play a causal role in the development of anxiety. Experimental studies also indicate a social learning component in anxiety via modelling (de Rosnay et al. 2006) and the transfer of threat-relevant information (Field et al. 2008). Social cognitive theory suggests that regardless of the initial cause of children’s anxiety, significant adults’ perceptions of threat, and their perception of the child’s ability to cope with threat can be reflected in adult-child interactions (e.g., warnings of danger, reinforcement of avoidant behaviours), which may help sustain an appraisal style characterised by threat and vulnerability (Barlow 1988; Creswell et al. 2011; Sharp et al. 2008). Evidence suggests a small but significant association between parenting behaviours and anxiety (McLeod et al. 2007).

## Anxiety in Autistic Children

As well as being highly prevalent (Simonoff et al. 2008), there is evidence to suggest there may be phenomenological differences in the anxiety experienced by autistic compared to non-autistic individuals. Kerns et al. (2014) identified both ‘traditional’ (DSM-defined) and what they described as ‘atypical’ anxiety in a sample of autistic children. The latter type of anxiety was defined as “exacerbated and clinically impairing anxiety around the hallmark features of ASD” (p. 2853) and exemplified by anxiety in relation to minor changes in schedule, unusual specific fears (e.g., the ‘happy birthday’ song) and social fearfulness in the absence of social judgment. Indeed, research into the etiology of anxiety in autistic children has tended to focus on the interplay between autistic symptoms such as social communication difficulties or sensory sensitivities, and anxiety symptoms (Bellini 2004; Duvekot et al. 2018; Green et al. 2012; Sukhodolsky et al. 2008). There is also a burgeoning literature on the role that cognitive mechanisms associated with autism such as intolerance of uncertainty (South and Rodgers 2017), emotional acceptance and alexithymia (Maisel et al. 2016) may play in the development of autistic children’s anxiety.

A multi-informant approach is recommended for assessing children’s mental health yet our understanding of anxiety in autistic children is largely based on parent-reported data (Van Steensel et al. 2011). Although concerns have been raised regarding the reliability of self-reported data on internalizing symptoms in autistic youth, for example due to poor concordance with diagnoses made through structured diagnostic interviews with parents (Mazefsky et al. 2011), a lack of agreement between informants on children’s internalizing symptoms is not unique to families with an autistic child, as evidenced by Achenbach et al. (1987) and more recently, De Los Reyes et al. (2015) (although see Cole et al. 2000; Nauta et al. 2004, for examples of concordance). Limitations to parent-report, as well as child-reported data have also been recognized, for example by Teagle (2002), who reported that only 39% of parents whose children met criteria for a psychiatric diagnosis perceived their child as having a problem. In children on the autistic spectrum, the possibility of false comorbidity adds complexity to this issue, with debate over whether core autism symptoms such as restricted and repetitive behaviours and social communication difficulties, are distinct from, or misinterpreted as, signs of anxiety (Kerns and Kendall 2012; Wood and Gadow 2010). When considered alongside evidence that non-autistic individuals find it particularly hard to interpret the mental states of those who are autistic (Brewer et al. 2016; Heasman and Gillespie 2017; Sheppard et al. 2015) and that some autistic individuals have identifying and describing their emotions (Maisel et al. 2016), it becomes clear that reliable self-report measures of cognitive elements of anxiety such as worry and intrusive thoughts (APA 2013), as well as the beliefs, assumptions and thinking styles that may accompany, generate or perpetuate anxiety are crucial.

Two studies have investigated autistic children’s interpretations as they relate to anxiety. Hollocks et al. (2016) reported that a group of 10- to 16-year-old boys with a diagnosis of both autism and one or more anxiety disorders were significantly more likely to choose threatening interpretations of ambiguous scenarios than a group of typically developing boys. The groups, however, were not matched on IQ and there were no differences in interpretations between the autistic adolescents with a co-occurring anxiety disorder and autistic adolescents without any such disorder. Sharma et al. (2014) reported that in response to hypothetical frustrating, rather than ambiguous, scenarios (developed by the authors based on past emotional experiences of the participants), autistic children aged 8–12 years perceived themselves as more accountable for the negative situation and less able to cope than a group of typical children of similar age and verbal ability. These studies provide suggestive evidence that cognitive biases might accompany anxiety in autistic children.

Parents of autistic children also experience greater levels of anxiety than their typical counterparts (Hayes and Watson 2013; Bitsika and Sharpley 2004). Proposed explanations for this difference include a lack of reciprocation from children in social interactions (Davis and Carter 2008), co-occurring externalizing symptoms in children (Lecavalier 2006) and a lack of social support (Lamminen 2008). Such factors could theoretically engender a sense of increased vulnerability among parents which then generalizes into an interpretative style. Yet, an association between parental anxiety and an interpretative style characterized by threat and vulnerability has hitherto not been investigated. As well as being relevant to the design of appropriate support for parents, there is some longitudinal evidence from a typical population of families to suggest that parental expectations of children’s coping abilities can play a causal role in the development of children’s anxiety over time (Creswell et al. 2011). It is theorized that poor parental expectations may lead to parenting practices that inadvertently increase children’s anxiety such as parental overprotection and excessive warnings of environmental threat (Creswell et al. 2010; Field and Lester 2010). Parents’ own interpretative style is also relevant as it may affect the way that they perceive and then report on their children’s current levels of anxiety (Bitsika et al. 2015).

In the current study, we administered the Ambiguous Scenarios Interview (Barrett et al. 1996; Creswell et al. 2014) to autistic children and their parents, and a comparison group of typical children and their parents. Our aims were threefold. First, we examined autistic children’s anxiety symptoms and their interpretations of ambiguous scenarios, as assessed through parent and child report, relative to typical children. Second, we examined anxiety and interpretations of adult-relevant ambiguous scenarios in mothers of autistic and typical children. Finally, we investigated the relationship between mothers’ and children’s anxiety and interpretations, as assessed through self and parent-report.

We hypothesised that anxiety symptoms in autistic children would be associated with negative interpretations of ambiguity as has been found previously in anxious children; that mothers of autistic children would also exhibit negative interpretations of ambiguity, and that these would extend to their interpretation of their child’s environment; and that mothers and children’s anxiety and responses to ambiguity would be positively associated.

## Methods

### Participants

Forty-seven cognitively able (verbal IQ > 70) children took part in this study, including 22 autistic children (18 boys) and 25 typical children (16 boys) aged between 6 and 14 years. Participants were recruited through the Autism Spectrum Database-UK (http://www.ASD-UK.com), mainstream and special schools, advertisements and parent support groups. Children in the two groups did not differ significantly in terms of age, t(45) = .91, *p* = .37, *d* = .27, gender, t(45) = 1.36, *p* = .17, *d* = .41 or verbal IQ, t(45) = 1.23, *p* = .23, *d* = .36 as assessed by the Wechsler Abbreviated Scales of Intelligence, Second Edition (WASI-II, Wechsler 2011) (see Table 1).

**Table 1:** Sample characteristics for the autistic and typical children

Measures	Autistic children (n = 22)	Typical children (n = 25)
Age		
M	10.24 (2.12)	9.68 (2.09)
Range	7.14–14.61	6.30–13.52
Verbal IQ^a^		
M	101.55 (14.57)	106.20 (11.39)
Range	76–129	87–137
SCQ^b^		
M	20.23 (6.89)	2.10 (2.60)
Range	8–33	0–8
ADOS-2^c^		
M	10.75 (3.43)	
Range	7–20	

^a^Verbal IQ was measured using the Wechsler Abbreviated Scales of Intelligence-2nd edition (Wechsler 2011). Standard scores are reported here (population M = 100, SD = 15)

^b^Social Communication Questionnaire (Rutter et al. 2003). A score of 15 or above indicates elevated levels of autistic symptomology

^c^ADOS-2 = Autism Diagnostic Observation Schedule-2nd edition (Lord et al. 2012). Scores of 7 and above indicate the presence of an autism spectrum disorder

All autistic children had previously received an independent clinical diagnosis of an autism spectrum condition according to ICD-10 (World Health Organisation 1993) or DSM-IV (American Psychiatric Association 2000) criteria and further met criteria on the Autism Diagnostic Observation Schedule (ADOS-G: Lord et al. 1999, 2012; ADOS-2:) using the revised algorithm (Gotham et al. 2007, 2008). All parents completed a screening measure for autism, the Social Communication Questionnaire (SCQ: Rutter et al. 2003). All children in the typical group scored well below the cut-off for autism (score of 15; see Table 1).

We also interviewed a subsample of 17 mothers of autistic children and 15 mothers of typical children. Their children were of similar age, t(30) = .92, *p* = .37, *d* = .32, gender, t(27.64) = 1.36, *p* = .19, *d* = .47 and verbal IQ, t(30) = 1.45, *p* = .16, *d* = .51.

### Measures

#### The Spence Children’s Anxiety Scale (SCAS; Spence 1998): Child-Self Report and Parent-Report

The SCAS is a multidimensional measure developed to assess subtypes of anxiety described in the DSM-IV (APA 1994), including generalised anxiety, panic/agoraphobia, separation anxiety, obsessive compulsive symptoms and physical injury fears. Respondents are asked to rate the frequency of anxiety item on a 4-point Likert scale ranging from 0 (never) to 3 (always). Responses to each of the 38 items are summed to create a total score, ranging from 0 to 114. The SCAS has good psychometric properties in non-autistic samples (Orgilés et al. 2016), including good test–retest reliability (Spence 1997), internal consistency (α = .92, Muris et al. 2000) and convergent validity (Brown-Jacobsen et al. 2011; Chorpita et al. 2000). The SCAS has also shown to have good internal consistency in autistic children (Cronbach’s alpha = .90, Boulter et al. 2014). A parent-report version of the scale has also been shown to have good psychometric properties (Nauta et al. 2004), including in parents of autistic children (Wigham and McConachie 2014; Zainal et al. 2014). In the current sample, there was good internal consistency in both the self-report (autistic α = .85, typical α = .83) and parent-report (autistic α = .93, typical α = .72) versions of the scale. The SCAS was originally designed for children aged eight and above but has been successfully used with younger samples (e.g., Neil and Smith 2017). In the current study the internal consistency of the self-report version of the scale remained good even among children aged six and seven (n = 7, Cronbach’s alpha = .79).

#### Beck Anxiety Inventory (BAI; Beck et al. 1988): Parent Self-Report

The BAI is a 21-item measure of adult anxiety. Participants are asked to indicate to what extent they have been bothered by each symptom over the past month, on a scale ranging from 0 (not at all) to 3 (severely). The test has been shown to have good psychometric properties including excellent internal consistency (α = .92) and test–retest reliability (*r* = .75 over 1 week) (Beck et al. 1988). In the current sample, the internal consistency of the scale was good for mothers of autistic children (α = .88) and acceptable for mothers of typical children (α = .61).

#### Ambiguous Scenarios Interview: Child Self-Report

The Ambiguous Scenarios Interview (adapted by Creswell et al. 2014; from Barrett et al. 1996) features 12 hypothetical scenarios, including six social (e.g., *“*You see a group of children from another class playing a great game. When you walk over to join them they are laughing”) and six non-social scenarios (e.g., “On the way to school you start to feel sick in the tummy”). Children are asked to (a) rate their level of distress in response to the scenario (“How upset would you feel in this situation?”; 0 = *not at all upset;* 10 = *very upset*), (b) give an open choice interpretation of the situation (e.g., “Why do you think they are laughing?”) (c) rate their perceived control over the situation *(*“How much would you be able to do about the situation”; 0 = *nothing*, 10 = *a lot)*, (d) offer an open choice behavioural response (“What would you do in this situation?”) and finally (e) make a choice between two interpretations (e.g., telling a nasty joke about you/laughing at the game): one threatening (coded as 1) and one non-threatening (coded as 0). In this study, open choice responses for (b) were coded as threatening or non-threatening (0 = non-threat, 1 = threat) and for (d) as avoidant or non-avoidant (0 = non-avoidant, 1 = avoidant) by an undergraduate psychology student blind to group and symptom scores, in accordance with the coding scheme used by Waite et al. (2015) (obtained via personal communication with C. Creswell, 6th October, 2015). To confirm the reliability of the coding scheme, a second independent rater coded a random sample (n = 40, 80%) of responses. Intraclass correlations were excellent for both threat (ICC = .97) and avoidance (ICC = .96) codings. Total scores were created for each of the five variables by summing the scores obtained in each of the 12 scenarios. Scores therefore ranged from 0 to 120 for negative emotions and perceived control, and from 0 to 12 for avoidant and threat responses. Higher scores indicate greater levels of negative emotions, perceived control, avoidance and perceived threat.

Autistic children’s responses showed similar, acceptable, levels of internal consistency to those of typical children for distress (autistic α = .83, typical α = .78), open choice threat (autistic α = .67, typical α = .68), forced choice threat (autistic α = .78, typical α = .69) and perceived control (autistic α = .81. typical α = .81). Similar to Waite et al. (2015), internal consistency for avoidant responses was poor in both groups (autistic α = .46, typical α = .47), which is unsurprising given that such responses were rare (see Table 2).

**Table 2:** Means and standard deviations on study measures for the autistic and non-autistic groups

M (SD)	Anxiety	Ambiguous Scenarios Interview Measures			
	SCAS^a^/BAI^b^	Threat^c^	Distress^c^	Control^c^	Avoidance^c^
Child–child					
Autistic					
M	29.67 (14.95)^d^	8.68 (5.51)^e^	51.77 (26.06)^e^	52.81 (25.81)^e^	1.32 (1.32)^e^
Range	5–61	1–21	12–101	20–103	0–4
Typical					
M	25.36 (10.68)^f^	7.04 (4.30)^g^	61.80 (21.23)^g^	48.40 (22.08)^g^	1.04 (1.21)^g^
Range	7–47	1–16	21–100	5–92	0–4
Mother–child					
Autistic					
M	28.70 (16.53)^h^	13.42 (4.20)^i^	70.88 (16.59)^i^	41.82 (24.04)^i^	4.41 (2.09)^i^
Range	6–66	7–22	45–102	7–113	1–8
Typical					
M	12.20 (5.81)^j^	7.80 (3.91)^k^	52.20 (20.22)^k^	60.00 (21.64)^k^	1.00 (.93)^k^
Range	1–25	3–16	13–89	33–96	0–3
Mother–mother					
Autistic					
M	12.89 (8.60)^l^	8.13 (3.79)^m^	59.88 (19.50)^m^	55.81 (17.04)^m^	44 (.81)^m^
Range	0–27	2–14	28–100	30–83	0–3
Typical					
M	5.21 (3.64)^n^	3.80 (3.00)^o^	42.80 (20.05)^o^	77.33 (23.86)^o^	.20 (.41)^o^
Range	0–12	1–11	12–78	38–114	0–1

^a^SCAS = Spence Children’s Anxiety Scale. Higher scores reflect greater levels of anxiety. ^b^BAI = Beck Anxiety Inventory. Higher scores reflect greater levels of anxiety. ^c^Ambiguous Scenarios Interview Measures. Greater scores reflect greater perceived threat, distress, control and avoidance

^d^n = 21, ^e^n = 22, ^f^n = 22, ^g^n = 25, ^h^n = 20, ^i^n = 17, ^j^n = 20, ^k^n = 15, ^l^n = 16, ^m^n = 16, ^n^n = 14, ^o^n = 15

In both groups, children’s open choice and forced choice threat responses were strongly correlated (autistic r = 80, *p* < .01; typical r = .55, *p* < .01) and in line with coefficients reported in previous studies (Creswell et al. 2006, 2014). These two variables were therefore combined, reducing the overall number of variables to four.

#### Ambiguous Scenarios Interview: Parent Report (About Their Child)

We also presented parents with the same 12 scenarios and asked them (a) to rate how distressed they perceived their child would be in response to each of them *(*0 = *not at all upset;* 10 = *very upset)*, (b) to give an open choice interpretation of the scenario from their child’s perspective e.g., “Why would X think they are laughing?”) (*open choice*); (c) to rate their own perception of their child’s control over the situation (“How much do you think your child can do about this?”) *(*0 = *nothing at all;* 10 = *a lot)*, and (d) to predict an open choice behavioural response for their child (e.g., “What will X do about it?”). As before, open choice responses were coded for threat and avoidance, according to Waite et al.’s (2015) coding schemes. A second independent rater coded a random sample of responses (48.6% of sample). Intraclass correlations were excellent for both threat (ICC = .97) and avoidance (n = .97) codings.

#### Ambiguous Scenarios Interview: Parent Self-Report

An adult version of the ambiguous scenarios interview (Creswell et al. 2005) featuring 12 adult-relevant ambiguous scenarios (e.g., “Not long after starting your new job your boss asks to see you”) was also presented to parents. Interviews followed the same format as the child self-report and parent-report versions, with parents asked (a) to rate their own distress in response to the scenarios on scales from 0 to 10, (b) to provide open-choice interpretations of what had happened (coded as 0 = non-threat, 1 = threat), (c) to rate how much control their child would have over the situation, (d) to offer a behavioural response (coded as 0 = non-avoidant, 1 = avoidant) and (e) to choose between a non-threatening (e.g., “they want to make sure you have settled in alright”, coded as 0) and threatening (“you haven’t been doing the job properly”, coded as 1) interpretation of the scenario. Again, in a sample of the responses (47%) intraclass correlations were excellent for both threat (ICC = .94) and avoidance (ICC = 1.0) responses.

### Procedure

Parents and their children attended a 45-min session within a university setting. Children were administered the Ambiguous Scenarios Interview and the Spence Children’s Anxiety Scale in a one-on-one session with a researcher. To check children’s understanding of the interview’s 0–10 response options, a practice question was given to children before the interview started. Children were probed for an example of something they enjoyed doing, and were then asked to describe on a scale from 0 to 10 how upset they would be if they were no longer allowed to participate in that activity. Once the interviewer was confident the child could use the response options, the interview proceeded.

Parents were interviewed separately, also in a one-on-one session with a researcher. In their session, parents were administered the parent-report and self-report versions of the Ambiguous Scenarios Interview. Following completion of the interviews, mothers were asked to complete the SCAS about their child and the BAI about themselves. Children completed the SCAS about themselves. Mother and child were then debriefed about the aims of the study.

Children were seen in an additional face-to-face session at the University where they were administered the verbal subscales of the Wechsler Abbreviated Scales of Intelligence, Second Edition (WASI-II; Wechsler 2011), and, for autistic children, the Autism Diagnostic Observation Schedule (ADOS-2; Lord et al. 1999, 2012). Parents were also given the Social Communication Questionnaire (SCQ: Rutter et al. 2003).

Ethical approval for this study was granted by the university’s research ethics committee. Parents provided written informed consent for their own and their child’s participation. Verbal assent was provided by participating children.

## Results

First, we begin by reporting analyses examining differences in autistic and non-autistic children’s anxiety/interpretations of ambiguity, as assessed through self and parent-report, and associations between anxiety and interpretations of ambiguity by group. Next, we report differences in self-reported anxiety/interpretations of ambiguity between *mothers* of autistic and non-autistic children, and associations between anxiety and interpretations of ambiguity by group. These analyses are followed by correlational analyses testing the associations between mothers’ and childrens’ anxiety/interpretations of ambiguity.

### Autistic and Non-autistic Children’s Anxiety and Responses to Ambiguity

Descriptive statistics are shown in Table 2. An independent samples t-test revealed no significant group difference in total child-reported SCAS scores, t(41) = 1.09, *p* = .28, *d* = .33, but parents rated their autistic children as significantly more anxious than typical children on the parent version of the SCAS, t(38) = 4.21, p < .001, *d* = 1.33. It is noteworthy that autistic children, typical children and parents of autistic children all reported similar amounts of child anxiety on the SCAS, whereas parents of typical children reported approximately half as much anxiety in their children. This pattern remained when the analysis was repeated to include only families where both the mother and child completed the SCAS (families with a typical child: n = 19, self-report SCAS: M = 12.31, SD = 5.94; M parent-report SCAS: M = 25.79, SD = 10.88. Families with an autistic child: n = 19, self-report: M = 30.26, SD = 15.62, parent-report M = 28.79, SD = 16.98).

To measure the impact of group on children’s four responses to ambiguity, we used the Bonferroni adjusted alpha level (0.05/4 = .013). A one-way MANOVA (using Pillai’s trace) revealed no significant differences between autistic and typical children’s self-reported responses to questions on threat, distress and control in the ambiguous scenarios, V = .11, F(3,43) = 1.85, *p* = .15, η_p_^2^ = .11. As children’s responses to the avoidance question were not normally distributed, a non-parametric test was carried out to compare the groups on this measure. A Mann–Whitney test revealed no significant difference in levels of self-reported avoidance between the autistic (Mdn = 1) and typical (Mdn = 1) children, U = 308, *z* = .74, *p* = .46, r = .11. We also examined responses to the social and non-social scenarios separately. One-way MANOVAs revealed no significant differences between autistic and typical children’s self-reported responses to either the social ambiguous scenarios, V = .12, F(4,42) = 1.45, *p* = .23, η_p_^2^ = .12, or the non-social scenarios, V = .15, F(4,42) = 1.81, *p* = .14, η_p_^2^ = .15.

There was, however, a significant effect of group on mothers’ responses, V = .59, F(4,27) = 9.70, p < .001, η_p_^2^ = .59. Mothers of autistic children reported that their children would perceive more threat in the scenarios, F(1,30) = 15.16, p = .001, η_p_^2^ = .34, behave in a more avoidant way, F(1,30) = 33.89, p < .001, η_p_^2^ = .53, and be more distressed, F(1,30) = 8.24, p = .007, η_p_^2^ = .22. They did not report that their child would be less in control, F(1,33) = 5.00 p = .03, η_p_^2^ = .14, than parents of typical children did.

To test the associations between children’s anxiety and responses to ambiguity, we used Bonferroni adjusted alpha level (0.05/4 = .013). There was a modest (but non-significant) positive association between autistic children’s reports of how distressed they would be in response to the ambiguous scenarios and their SCAS scores (r = .45, *p* = .04). A similar, but weaker, relationship was found in the typical group (r = .34, *p* = .13). Contrary to predictions, associations between children’s self-reported anxiety and their threat interpretations, perceived avoidance or perceived control were not significantly associated in either the autistic or typical group (see Table 3). Again, using Bonferroni adjusted alpha level (0.05/4 = .013), there was a significant positive association between mothers’ reports of their autistic children’s anxiety and mothers’ reports of how distressed their child would be in an ambiguous scenario (r = .67, *p* = .01). There was also a medium to strong (but non-significant) association between mothers’ reports of their autistic children’s anxiety and mothers’ reports of how avoidant their children would be (r = .60, *p* = .02). This pattern was not reflected in the typical group, among whom there was only one notable association, a negative (but not significant) relationship between anxiety and perceived control (r = − .60, *p* = .02).

**Table 3:** Correlations between interview and anxiety measures

	1	2	3	4	5	6	7	8	9	10	11	12	13	14	15
Child–child															
1. Anxiety		.22	.34	− .24	.16	.34	.48	.20	− .41	.50	.35	− .01	.26	.10	.37
2. Threat	.12		.13	− .04	.43*	− .09	.24	.06	− .08	.12	.25	.01	.05	.48	.13
3. Distress	.45*	.46*		− .11	− .01	.10	.09	− .19	.15	− .02	− .19	.09	.01	.12	.33
4. Control	.01	.45*	.66**		− .45*	− .20	.11	.33	.78**	.14	− .39	.42	.43	.36	− .49
5. Avoidance	.16	.61**	.51*	.36		− .07	.08	− .15	− .45	.19	.39	− .42	− .15	.20	.58
Mother–child															
6. Anxiety	.60*	.14	.24	.2	.17		.10	− .16	− .60*	− .11	.40	.40	− .03	− .55*	.27
7. Threat	.17	.06	− .04	.01	.3	.39		.65**	.19	.53*	.04	.10	.53*	.15	.03
8. Distress	.19	.12	− .02	.16	.29	.67**	.78**		.48	.40	.28	.29	.83**	.14	− .52*
9. Control	− .13	− .28	− .44	− .10	− .35	.36	− .03	.19		.14	− .45	.12	.35	.37	− .49
10. Avoidance	.59*	− .21	.16	− .11	.18	.60*	.61*	.54*	.08		− .22	− .03	.44	.20	.061
Mother–mother															
11. Anxiety	.17	.17	.11	.24	.21	.78**	.58*	.80**	.21	.42		.22	.29	− .35	.21
12. Threat	− .06	− .19	.06	.24	.04	.52	.32	.53*	.37	.14	.60*		.54*	− .14	− .37
13. Distress	− .05	− .15	.10	.43	.05	.60	.29	.60*	.39	.13	.59*	.90**		.10	− .38
14. Control	.12	− .32	− .18	.02	− .31	.20	.34	.17	.60*	.46	.02	.14	.14		− .25
15. Avoidance	.60*	− .003	.18	− .09	− .06	.72**	.10	.34	.22	.65**	.46	.18	.20	.01	

Autistic children’s and their parents’ data is below the diagonal, typical children’s and their parents’ data is above the diagonal

*p < .05, **p < .01

### Anxiety and Responses to Ambiguity in Mothers of Autistic and Non-autistic Children

Mothers of autistic children reported significantly greater levels of anxiety in themselves, t(28) = 2.88, *p* = .007, *d* = .43, than mothers of typical children. A one-way MANOVA using Pillai’s trace also revealed a significant effect of group on mothers’ own reported responses to ambiguity, V = .42, F(3,27) = 6.42, *p* = .002, η_p_^2^ = .42. Using the Bonferroni adjusted alpha level (0.05/4 = .013), mothers of autistic children perceived more threat to themselves in the ambiguous scenarios, F(1,29) = 12.27, *p* = .002, η_p_^2^ = .30, and reported being less in control, F(1,29) = 8.44, *p* = .007, η_p_^2^ = .23, than their typical counterparts, however, differences in reports of how distressed they would feel did not reach significance, F(1,29) = 5.78, *p* = .023, η_p_^2^ = .17. Using a non-parametric test, there was no significant difference in how avoidant mothers of autistic children (Mdn = 0) compared to mothers of typical children (Mdn = 0) reported they would be in the adult-referent scenarios, U = 135, *z* = .78, p = .57, r = .14, with very few predictions of avoidant behaviour among either group.

To examine the association between mothers’ anxiety and their responses to ambiguity, we once again used a Bonferroni-adjusted alpha level of .013. In the autistic group (n = 16), mothers’ self-reported anxiety was positively yet marginally associated with their own perceptions of threat (r = .60, *p* = .014) and distress (r = .59, *p* = .016). In the typical group (n = 14), there were no significant associations (all *p*s > .21).

### Associations Between Mothers’ and Children’s Anxiety and Responses to Ambiguity

We hypothesised that maternal anxiety and responses to ambiguity would be associated with children’s self-reported anxiety and responses to ambiguity. There was no significant association between mothers’ and children’s self-reported anxiety in either group (autistic r = .17, *p* = .53; typical r = .35, *p* = .22). Nor were there were significant associations between maternal-threat and child-threat, maternal-distress and child-distress, maternal-control and child-control or maternal-avoidance and child-avoidance in either group (all rs < .36, all *p*s > .19).

There were, however, significant associations between mothers’ own anxiety and interpretation biases and mothers’ reports of their children’s anxiety and interpretation biases. In these accounts, mothers’ anxiety was very strongly associated with children’s anxiety in the autistic group (r = .78, *p* = .001) but less so in the typical group (r = .40, *p* = .16) (see Table 3). A similar pattern was observed for avoidance, where a non-parametric Spearman’s rho correlation coefficient was used, (autistic r_s_ = .65, *p* = .007; typical r_s_ = .061, *p* = .83) and control (autistic r = .60, *p* = .014; typical r = .37, *p* = .18). Mothers’ own distress and mothers’ reports of children’s distress were associated in both groups (autistic r = .64, *p* = .007; typical r = .83, *p* = .003).

## Discussion

Numerous studies have shown that autistic children, and their mothers, have atypically high levels of anxiety, yet little research has investigated the beliefs, assumptions and thinking styles traditionally associated with anxiety in non-autistic populations and targeted by cognitive behavioural therapy (Beck et al. 1985). We investigated anxiety and interpretation biases, as assessed by responses to ambiguity, in cognitively able autistic children, typical children, and their mothers. Autistic children self-reported similar interpretations of ambiguity, and similar levels of anxiety, to their typical peers. In contrast, mothers of autistic children reported more negative interpretations of ambiguity and anxiety in both their children and themselves, relative to mothers of typical children. Within our relatively small sample, children’s self-reported anxiety was not significantly associated with their interpretations of ambiguity in either autistic or typical children. In contrast, mothers’ reports of children’s anxiety and mothers’ reports of children’s interpretations of ambiguity showed a stronger pattern of association, albeit only in the autistic group. Mothers’ own anxiety was also more strongly associated with their reports of children’s anxiety and interpretations in the autistic group.

A cognitive appraisal style, characterised by threat and vulnerability, has been identified in anxious children (Barrett et al. 1996; Creswell et al. 2005; Waters et al. 2008). Unlike Hollocks et al. (2016), however, we found no evidence of an association between anxiety and threat interpretation in autistic children’s self-reports, or indeed in typical children. This is possibly due to differences in the age range of the children sampled, with children in our sample having a mean age of 10 years, compared to a mean of 13 years in Hollocks et al.’s sample. Creswell et al. (2014) and Waite et al. (2015) who, similarly, found no difference in threat interpretation in children with and without anxiety disorders, suggested that age may be a moderating factor in the relationship between anxiety and thinking styles. The idea that cognitive appraisals that maintain anxiety develop only later on in childhood is also supported by a recent review (Stuijfzand et al. 2017). Our data suggest there may be a similar developmental course for interpretation biases in autistic children. The only significant association between children’s anxiety and their responses to ambiguity was found in mothers’ accounts: mothers’ reports of children’s anxiety were associated with mothers’ reports of children’s hypothetical distress in the ambiguous scenarios. One possible interpretation of this finding is that mothers’ accounts of children’s anxiety are, in part, driven by children’s externalising symptoms (e.g., the extent to which they show visible distress), and that this distress is viewed by parents as a clearer indicator of children’s anxiety than children’s cognitions (e.g., their interpretation of threat).

The finding that autistic and typical children reported similar levels of ‘traditional’ (DSM-4 defined) anxiety and interpretation biases may seem surprising. Yet research into anxiety in autism is dominated by parent, rather than child report (Van Steensel et al. 2011), and here, as in previous studies (e.g., Boulter et al. 2014; Neil et al. 2016), mothers reported significantly more anxiety in their autistic children than parents of typical children. The possibility that autistic children are under-reporting their anxiety symptoms, perhaps due to difficulties recognising and labelling emotions, is not supported by our data, where autistic children reported similar levels of symptoms in themselves as their mothers reported in them. Rather, it was the typical group where differences in mother and child report were evident, with typical parents appearing to report fewer symptoms in their children. This is not the first time parents’ and children have appeared more compatible in terms of children’s anxiety levels in families with an autistic, as opposed to typically developing child (Boulter et al. 2014). It is possible that typical children use their abilities in social interaction and communication to mask their anxiety. Alternatively, parents of autistic children may rely on autistic or other co-occurring symptoms, rather than anxiety symptoms to make judgements regarding their children’s anxiety levels. The levels of anxiety reported by autistic (and typical) children, while similar to three other studies carried out in the UK (e.g., Neil and Smith 2017; Smith et al. 2017; Stallard et al. 2005) and the original data from Australia (Spence 1998), are actually quite high in comparison to those reported by children from some other countries (Essau et al. 2004; Muris et al. 2000), which further speak against the under-reporting of symptoms in the autistic group.

The measure of anxiety in this study covered ‘traditional’ (DSM-defined) rather than autistic-specific anxiety symptoms, which may to help explain the similarities in self-reported anxiety levels between the groups (Kerns et al. 2014). A more autism-relevant anxiety measure, such as that recently developed by Rodgers et al. (2016), which focuses on ‘sensory anxiety’, intolerance of uncertainty and phobias may have revealed greater differences.

While previous research has shown that mothers of autistic children can experience particularly high levels of stress (Hayes and Watson 2013; Dumas et al. 1991; Koegel et al. 1992) and anxiety (Bitsika and Sharpley 2004), our data also demonstrated a more negative interpretative style in this group. Importantly, this was evident both in situations that did and did not involve their children: mothers perceived more threat to themselves, and perceived themselves to be more vulnerable, even in situations which had nothing to do with their child (e.g., at work or at adult social events). Parental psychopathology has been linked to poorer treatment outcomes for anxiety in children (Hudson et al. 2014; Bodden et al. 2008), and there is some evidence that interventions for youth anxiety are more successful when parental anxiety is also treated (Cobham et al. 2010). A clinical implication of our study is that targeting parents’ own negative thinking styles may be a helpful addition to interventions for both children and parents.

Contrary to predictions, we found little evidence of an association between mothers’ anxiety/interpretation biases and children’s self-reported anxiety/interpretation biases. This speaks against the role of parenting factors in the development of children’s anxiety. We did, however, find a particularly strong association between mothers’ own anxiety and their reports of children’s anxiety and coping in the autistic group. A limitation of our cross-sectional data is that it cannot explain what is driving this association. It is possible, however, that, regardless of the cause, mothers’ negative interpretative style could come to influence the way they view and report their children’s anxiety and interpretations, which is particularly relevant given the reliance on parent-report data when it comes to autistic children’s anxiety (Storch et al. 2012). Poor expectations among significant adults may also lead to well-meaning protective behaviours such as the transfer of threat-relevant information (Muris and Field 2010) and reinforcement of avoidant responses (Barrett et al. 1996), which, in the longer term, may contribute to or maintain an appraisal style characterised by threat and vulnerability in later childhood or adolescence (Sharp et al. 2008). Alternatively, the association between mothers’ anxiety and their accounts of their children’s ‘traditional’ anxiety might be moderated by the severity of children’s autistic symptoms, or children’s qualitatively different anxiety symptoms. These symptoms (such as repetitive and restricted behaviours), may give rise to perceptions of these children as more traditionally anxious. This perception, combined with the potential stresses involved in parenting an autistic child, may lead to greater maternal anxiety. In line with this possibility, preliminary assessments of the efficacy of a newly-devised treatment for intolerance of uncertainty targeted at young people with autism spectrum disorder, but delivered by parents (Rodgers et al. 2017), indicated improvements in children’s (parent-reported) intolerance of uncertainty and also parents’ own stress symptoms.

Parents of typical children, in contrast, appeared to underestimate their children’s anxiety and overestimate their coping skills, compared to self-report. Bitsika et al. (2015) found that only reports from mothers who were above minimally-anxious were in line with clinicians’ reports of autistic boys’ generalised anxiety. One interpretation of our data is therefore that the lower levels of anxiety in parents of typically developing children translated to less hypervigilance to, or recognition of, children’s anxiety. In turn this may have resulted in a more positive interpretation of their children’s emotional states.

Our study is not without its limitations. First, it is possible that the sample size in combination with a conservative analytical approach may have precluded the finding of some significant differences and associations in the data. There are three occasions where hypothesised associations between children’s anxiety and interpretations do not reach significance due to our application of Bonferroni correction to deal with multiple comparisons. Given that these effect sizes are medium-strong in magnitude and theoretically interesting, it will be important for others to replicate them in future research with a larger sample size. That said, the sample size did not preclude the detection of a number of significant and clinically-interesting findings in the parents’ data, and the small effect sizes in the majority of associations between children’s self-reported anxiety and interpretations suggest that a larger sample size is unlikely to alter the pattern of results here. Second, our assessment of anxious symptoms, rather than anxiety disorders, was appropriate in this instance given both that anxiety disorders tend to be underdiagnosed (Chavira et al. 2004) and that anxiety causes impairment in those who do not reach criteria for a disorder (Angold et al. 1999). It is possible, however, that some interpretation biases are only evident in individuals with clinically-significant levels of symptoms. To address this issue specifically, future research should implement a two-by-two design including participants with above and below clinical levels of anxiety in both groups. Finally, the findings from our verbally and cognitively able group of autistic children may not translate to those with difficulties with language or other cognitive difficulties. Nevertheless, this study was original in its use of the ambiguous situations interview to measure a wide range of responses to ambiguity in autistic children. Future research should investigate whether mothers’ poor expectations of their children’s responses to ambiguity translate into parenting behaviours such as overprotection and warnings of threats and whether these inadvertently lead to increases in children’s anxiety levels.

In conclusion, this study is one of the only studies to have investigated interpretation biases as they relate to anxiety in autistic children. It appears that the differences between autistic and typical children’s anxiety and perceptions of coping ability are more evident in parent, than child report. It will be useful to establish how parental factors (e.g., a negative memory bias or negative interpretative style) and/or child factors (e.g., degree and nature of autistic and anxious symptomatology) contribute to this difference. Importantly, the study underlines the importance of extending the multi-informant approach to understanding anxiety to autistic children. Encouragingly, the similarities between autistic and typical individuals’ accounts of their anxiety and interpretations in childhood suggest an opportunity to prevent a negative interpretation bias emerging later in development.
